# Mitigation of salt stress in lettuce by a biostimulant that protects the root absorption zone and improves biochemical responses

**DOI:** 10.3389/fpls.2024.1341714

**Published:** 2024-02-16

**Authors:** Javier Zuzunaga-Rosas, Roberta Calone, Diana M. Mircea, Rashmi Shakya, Sara Ibáñez-Asensio, Monica Boscaiu, Ana Fita, Héctor Moreno-Ramón, Oscar Vicente

**Affiliations:** ^1^ Department of Plant Production, Universitat Politècnica de València, Valencia, Spain; ^2^ Innovak Global S. A. de C. V., La Concordia, Chihuahua, Mexico; ^3^ Council for Agricultural Research and Economics (CREA), Research Centre for Agriculture and Environment, Bologna, Rome, Italy; ^4^ Department of Forestry, University of Agricultural Sciences and Veterinary Medicine Cluj-Napoca, Cluj-Napoca, Romania; ^5^ Institute for the Conservation and Improvement of Valencian Agrodiversity (COMAV), Universitat Politècnica de València, Valencia, Spain; ^6^ Department of Botany, Miranda House, University of Delhi, Delhi, India; ^7^ Mediterranean Agroforestry Institute (IAM), Universitat Politècnica de València, Valencia, Spain

**Keywords:** climate change, sustainable agriculture, salt tolerance, nutrient absorption, protein hydrolysates, ion transport, osmolytes, antioxidant systems

## Abstract

Horticultural crops constantly face abiotic stress factors such as salinity, which have intensified in recent years due to accelerated climate change, significantly affecting their yields and profitability. Under these conditions, it has become necessary to implement effective and sustainable solutions to guarantee agricultural productivity and food security. The influence of BALOX^®^, a biostimulant of plant origin, was tested on the responses to salinity of *Lactuca sativa* L. var. *longifolia* plants exposed to salt concentrations up to 150 mM NaCl, evaluating different biometric and biochemical properties after 25 days of treatment. Control plants were cultivated under the same conditions but without the biostimulant treatment. An *in situ* analysis of root characteristics using a non-destructive, real-time method was also performed. The salt stress treatments inhibited plant growth, reduced chlorophyll and carotenoid contents, and increased the concentrations of Na^+^ and Cl^-^ in roots and leaves while reducing those of Ca^2+^. BALOX^®^ application had a positive effect because it stimulated plant growth and the level of Ca^2+^ and photosynthetic pigments. In addition, it reduced the content of Na^+^ and Cl^-^ in the presence and the absence of salt. The biostimulant also reduced the salt-induced accumulation of stress biomarkers, such as proline, malondialdehyde (MDA), and hydrogen peroxide (H_2_O_2_). Therefore, BALOX^®^ appears to significantly reduce osmotic, ionic and oxidative stress levels in salt-treated plants. Furthermore, the analysis of the salt treatments’ and the biostimulant’s direct effects on roots indicated that BALOX^®^’s primary mechanism of action probably involves improving plant nutrition, even under severe salt stress conditions, by protecting and stimulating the root absorption zone.

## 1 Introduction

It is projected that, by 2050, agricultural production will have to double to meet population growth, and this will increase the pressure on natural resources due to increased consumption of meat, fruits and vegetables (FAO, 2017). Agriculture also faces abiotic stress challenges intensified in recent years by global warming effects, such as temperature extremes, drought, salinity, environmental pollutants, and nutritional deficiencies. These constraints are especially severe for horticultural crops, which are generally more susceptible to altered environmental conditions (Walter et al., 2013; Dong et al., 2020).

Amongst environmental stresses, salinity is one of the most widespread and detrimental to the production of many crops; it is estimated that it will affect more than half of the irrigated land worldwide by 2050 (Bartels and Sunkar, 2005). Furthermore, over 25% of irrigated agricultural areas in Mediterranean countries are already salinised (Zia-ur-Rehman et al., 2016). All predictions point to annual increases in extension and severity of soil salinisation in arid and semi-arid regions (Stolte et al., 2016). This problem is further exacerbated by excessive use of saline irrigation water (Singh, 2022), mainly due to seawater intrusion in cultivated areas close to the sea, which has been reported to cause a 50% yield reduction in susceptible crops such as lettuce (Miceli et al., 2003). Generally, most horticultural crops are highly susceptible to salinity, with a threshold of no more than 2 dS m^-1^ (Maas and Hoffman, 1977).

Lettuce (*Lactuca sativa* L.), a horticultural crop of economic importance, is one of the *Asteraceae* family’s most extensively cultivated and eaten fresh vegetables. (Rubatzky and Yamaguchi, 1997). Its global production reached more than 29 million tonnes in 2019, with about one million produced in Spain. China is the leading grower, followed by the United States and India (FAO, 2023).

Modern lettuce cultivars, with a great diversity of morphological characteristics and many colours, are mainly classified into crisphead, butterhead, romaine, loose-leaf or stem lettuce (Zhang et al., 2017). Its consumption is increasing as it is low in calories and rich in fibre and is a source of bioactive components: various vitamins (B, C, E, K) and health-promoting phytochemicals, including phenolic compounds, carotenoids, ascorbic acid, or tocopherols. These metabolites are potent antioxidants, reported to possess anti-cancer, anti-diabetic, anti-inflammatory and anti-cardiovascular disease properties, also improving vitality and delaying ageing (Iakimova and Woltering, 2015; Kim et al., 2016; Chen et al., 2020). However, lettuce plants are relatively sensitive to salinity, with a tolerance threshold between 1.1 and 2.0 dS m^-1^ (Andriolo et al., 2005; Uenluekara et al., 2008).

Salinity causes a significant reduction in plant development and a loss of yield through ionic, osmotic and oxidative stress (Hasegawa et al., 2000; Munns and Tester, 2008). High sodium and chloride concentrations are toxic, inhibiting the uptake of essential nutrients by roots and limiting normal plant development (MaChado and Serralheiro, 2017). Furthermore, salinity can cause cellular dehydration, inhibition of enzyme activities and basic metabolic processes, direct inactivation of proteins, alteration of cellular homeostasis, and inhibition of photosynthetic activity while increasing the generation of reactive oxygen species (ROS) and lipid peroxidation (Forni et al., 2017).

Consequently, in an effort to feed a hungry and expanding global population (Alturki et al., 2020), it is necessary to develop agricultural technologies and practices to ensure food security, improving crop yields even under adverse environmental conditions. In this sense, biostimulants provide a new approach that modifies the physiological processes of plants. Biostimulants can induce an increase in yield, the stimulation of plant growth and the mitigation of stresses induced by human action or changing environmental conditions (Yakhin et al., 2017). Indeed, biostimulants have been considered sustainable solutions and advanced agronomic tools, as demonstrated by the rise in publications and their market’s consistent growth (Colla et al., 2015; Du Jardin, 2015; Povero et al., 2016). By 2027, the global biostimulants market will probably reach USD 9220 million (Ikuyinminu et al., 2022). Biostimulants include a varied collection of microorganisms and crude or purified substances, extracts and compounds: mycorrhizal fungi, nitrogen-fixing bacteria, humic acids, seaweed extracts, protein hydrolysates, phenolic compounds, glycine betaine, amongst others (Du Jardin, 2015; Rouphael and Colla, 2018). Protein hydrolysates (i.e., a mixture of amino acids, oligopeptides, and polypeptides) have been found to enhance plant development and productivity under environmental constraints such as salinity (Colla et al., 2015). However, the mechanisms activated by biostimulants are often challenging to identify due to their complex nature and are an ongoing subject of research (Paul et al., 2019).

This study aimed to test the effect of a plant-derived biostimulant (BALOX^®^) on the tolerance of lettuce plants (*Lactuca sativa* L.) to salt stress, evaluating biometric and biochemical responses triggered by the biostimulant. BALOX^®^ is based on glycine betaine and polyphenols and is obtained from rice husk and oat hydrolysates. The study also included the *in situ* analysis of root characteristics of plants grown under different NaCl concentrations by a real-time, non-destructive method. The starting hypothesis was that BALOX^®^ application in plants subjected to different NaCl concentrations could stimulate growth, increasing root area, mainly affecting the fine roots responsible for nutrient uptake.

## 2 Materials and methods

### 2.1 Plant growth, biostimulant and salt stress treatments

Romaine lettuce (*Lactuca sativa* L. var. *longifolia*) seeds were sown in a greenhouse at the Universitat Politècnica de València (Spain) in trays containing a commercial peat and vermiculite (3:1) mixture. Temperature (18-23°C) and relative humidity (50-70%) were controlled in a light/dark photoperiod of 16/8 hours. The seedlings (which had five true leaves) were moved into separate pots with a diameter of 12 cm and 450 g of the same substrate after 14 days. Subsequently, the pots were arranged in eight trays, each measuring 57 cm in length, 42 cm in width, and 9 cm in depth. The total number of plants in the study was 40, with five replicates (individual plants) for each treatment placed in a separate tray.

The experimental design consisted of the application of the biostimulant and testing its effect on plants subjected to increasing concentrations of NaCl in irrigation water: 0, 50 (5.36 dS m^-1^), 100 (10.50 dS m^-1^) and 150 mM (15.42 dS m^-1^) NaCl for 25 days. Before starting the salt treatments, plants were watered twice weekly with 0.15 L per pot of tap water (EC = 0.90 dS m^-1^).

The application of the biostimulant began on the 28^th^ of March, 2022, four days after transplanting. Each plant was given three BALOX^®^ applications with a dose of 0.6 mL L^-1^ (equivalent to 3 L per hectare, according to the manufacturing company indications) at 15-day intervals. The biostimulant is of plant origin, made from rice and oat husk protein hydrolysates, and formulated by Innovak Global SA (Chihuahua, Mexico). Apart from the complex mixture of amino acids and oligo peptides produced by protein hydrolysis, its main active components are polyphenols [1.4% (w/w), expressed as gallic acid equivalents], and glycine betaine [3.0% (w/w)], added to the product. BALOX^®^ biostimulant is a registered trademark and is applied to the root system by irrigation (alone or mixed with fertiliser); it is not intended for use on the leaves.

Salt treatments began 18 days after transplantation when the seedlings were fully established, coinciding with the second application of the biostimulant. The increasing NaCl concentrations were applied in such a way that two trays, with five pots each, were used for each salt treatment, one for plants with biostimulant application and one for control plants (without biostimulant). The plants were irrigated twice weekly with 0.15 L of salt solutions with the NaCl concentrations indicated above. The greenhouse experiment was finalised after 42 days from the transplant and 25 days of salt stress treatments on the 5^th^ of May, 2022, when plants were transported to the laboratory at the Institute for the Conservation and Improvement of Valencian Agrodiversity (COMAV) for further morphological and biochemical analysis.

### 2.2 Growth parameters

Several growth parameters were recorded at the end of the treatments, 42 days after transplantation. The roots and aerial parts were analysed independently. The roots were brushed to remove any traces of soil and were subsequently weighed with a precision scale. In this way, the fresh weight (FW) of roots and leaves was obtained. Both parts of the plant sample were divided into portions that were frozen in liquid nitrogen and kept at -75°C until the analysis. The leftover material was weighed (FW), dried for about 72 hours at 65°C, and then weighed to register the dry weight (DW). Finally, the following formula was used to determine each sample’s water content (WC):


$$WC\%=\left[\frac{FW-DW}{FW}\right]*100$$

### 2.3 Measurement of electrical conductivity

The EC_1:5_ of the substrate was determined in two periods: at the beginning and the end of the experiments. Five pots per treatment were used to determine the mean value, but before, samples were air-dried and passed through a 2 mm sieve. A suspension of the substrate in Milli Q water was prepared (1:5), stirred for half an hour and then filtered. The electrical conductivity (dS m^-1^) was measured with a Crison 522 conductivity meter (Crison Instruments, S.A., Barcelona, Spain).

### 2.4 Camera and software details for root observations

**Figure 1:**
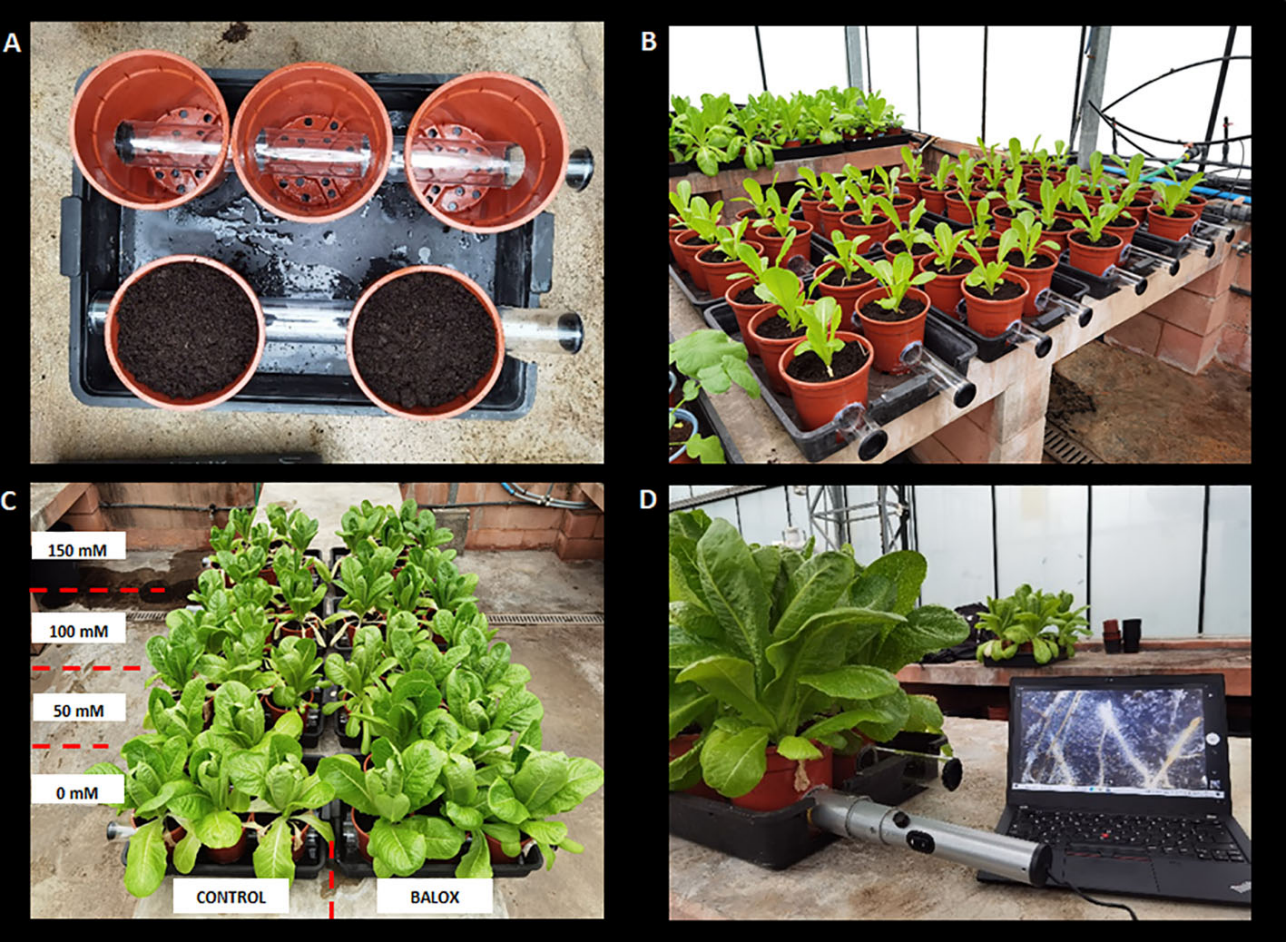
Setting up of rhizotron tubes and arrangement of pots in trays **(A)**, growth of plants under treatments with different concentrations of NaCl **(B)**, plants at the end of treatments, 42 days after transplanting and cutting of lettuce plants **(C)**, real-time root evaluation with Microrhizotron camera model ST 21 **(D)**.

To determine the effect of salinity and the impact of the biostimulant on the roots of lettuce plants, 16 transparent acrylic tubes, i.e., two tubes per tray (62 cm long x 6 cm in diameter), were used. Two circular holes (6.2 cm in diameter) were made 3 cm above the base of each pot to enter the tubes, as shown in **Figure 1A**. In each tray, five pots were arranged in two rows (3 + 2). Therefore, two holes per pot row in line with the holes of pots were also made in each tray. After correctly placing the tube through the holes in pots, the hole was sealed with liquid silicone to avoid the entry of light and air and the exit of substrate and water. The ends of the tubes were sealed with rubber caps until root evaluation was done using the minirhizotron chamber (**Figure 1**).

The observation of roots for all salt and biostimulant treatments was done three days before harvesting the plants. For this purpose, the Microrhizotron camera, ST 21 model manufactured by Innovak Global S.A de C.V. (**Figure 1D**), was used. It consists of a cylindrical structure (diameter: 4.5 cm and length: 79 cm) having a dual axis microscope lens (10x magnification and continuous magnification up to 300x), 2 MP image sensor, adjustable light intensity, and shutter range of 1 to 1/1000 sec. The camera facilitates shooting 360 degrees and has a USB cable with viewing software. To calculate root surface area (cm^2^), the software WinRhizo Pro (WinRhizo Pro 2003b, Reagent Instruments Inc. Quebec, Canada) was used.

### 2.5 Quantification of photosynthetic pigments

Total chlorophylls (tChl) and carotenoids (Caro) were quantified spectrophotometrically according to Lichtenthaler and Wellburn (1983). Pigments were extracted with 1 mL of 80% (v/v) ice-cold acetone from freshly ground leaf samples (50 mg). The samples were shaken at 4°C for 12 h in darkness, followed by centrifugation at 13,300 x *g* for 10 min at 4°C. The supernatant was collected, and the absorbance was measured at 663, 646 and 470 nm. The calculated concentrations were expressed in mg g^-1^ DW.

### 2.6 Quantification of ions

Na^+^, K^+^, Cl^-^ and Ca^2+^ concentrations in roots and leaves were determined separately from ground, dried samples according to Weimberg (1987). The aqueous extracts were prepared by mixing ground dried samples (100 mg) with 2 mL of deionised water, followed by incubation in a water bath at 95°C for 1 h. Samples were cooled on ice, mixed overnight on a shaker, centrifuged at 13,300 x *g* for 10 min, and the supernatant was collected to determine ion contents. Cation contents were measured with a PFP7 flame photometer (Jenway Inc., Burlington, VT, USA), and Cl^-^ with an MKII Chloride Analyser 926 (Sherwood, Inc., Cambridge, UK).

### 2.7 Quantification of osmolytes

Leaf samples were analysed to determine three plant osmolytes: proline (Pro), glycine betaine (GB) and total soluble sugars (TSS). The acid ninhydrin method was used for quantifying Pro content (Bates et al., 1973). Freshly ground samples (50 mg) were mixed with 1 mL of a 3% (w/v) aqueous sulphosalicylic acid solution and centrifuged at 13,300 x *g* for 15 min at room temperature. The supernatant was mixed with ninhydrin and acetic acid, incubated at 95°C for 1 h, cooled on ice and extracted with two volumes of toluene. The absorbance of the collected organic phase was measured at 520 nm using toluene as the blank. Samples with known Pro concentrations were assayed in parallel to obtain a standard curve, and proline concentrations were expressed in µmol g^-1^ DW.

GB was quantified according to Grieve and Grattan (1983) with slight modifications (Zuzunaga-Rosas et al., 2022). Freshly ground samples (150 mg) were mixed with 1.5 mL of Milli Q water, kept on a shaker for 24 h at 4°C and centrifuged at 13,300 x *g* for 10 min at 0°C. The supernatant was collected, mixed with 2 N sulphuric acid solution (1:1) and incubated on ice for 1 h. To 125 µl of this sample, 50 µl of ice-cold KI-I_2_ solution was added (to induce precipitation of GB in golden crystals form), and samples were incubated for 16 h at 4°C followed by centrifugation at 13,300 x *g* for 45 min at 0°C. The supernatant was carefully removed, and GB crystals were dissolved in 1.4 mL of ice-cold 1, 2-dichloroethane. Samples were incubated for 2.5 h at 4°C in darkness, and the absorbance was measured at 365 nm. The GB concentrations were calculated in µmol g^−1^ DW.

TSS contents in samples were determined according to Dubois et al. (1956). Freshly ground leaves (50 mg) were extracted with 80% methanol (v/v) on a shaker for 24 h at 4°C and centrifuged at 13,300 x *g* for 10 min at 0°C. Phenol (5%, v/v) and concentrated sulphuric acid were added to the supernatant, and the sample was incubated for 20 min at room temperature. The absorbance was measured at 490 nm, and TSS concentrations were expressed in equivalents of the glucose used as standard (mg eq. glucose g^-1^ DW).

### 2.8 Determination of oxidative stress biomarkers

Hydrogen peroxide (H_2_O_2_) and malondialdehyde (MDA) levels were determined to evaluate the extent of oxidative stress in the samples. H_2_O_2_ determination was carried out according to Loreto and Valikova (2001). Samples (50 mg) were extracted with 500 µL of 0.1% (w/v) trichloroacetic acid (TCA) followed by centrifugation at 13,000 x *g* at 4°C; 500 µL potassium phosphate buffer (10 mM, pH 7.0) and 1 mL of KI (1 M) were then added to the supernatant. The samples were incubated for 1 h in darkness at room temperature (RT), and the absorbance was measured at 390 nm. H_2_O_2_ concentrations were expressed as µmol g^−1^DW.

MDA quantification followed the protocol by Hodges et al. (1999) with some modifications (Taulavuori et al., 2001). Methanolic extracts of the samples (the same used for TSS measurements) were mixed with 0.5% (w/v) thiobarbituric acid (TBA) dissolved in a 20% (w/v) TCA solution. The blank for each sample was prepared separately by mixing the extract with 20% TCA. The samples were incubated at 95°C for 20 min, followed by cooling on ice to stop the reaction and then centrifuged at 13,300 x *g* for 10 min at 4°C. Finally, the supernatant absorbance was measured at 440, 600 and 532 nm. MDA content was expressed as nmol g^−1^ DW.

### 2.9 Antioxidant compounds quantification

Flavonoids (TF) and total phenolic compounds (TPC) were determined using the methanolic extracts described above. TPC contents were determined according to Blainski et al. (2013) using the Folin-Ciocalteu reagent (FCR). Samples (100 µL) were mixed with 100 µL of FCR and 350 µL of 15% (w/v) Na_2_CO_3_, followed by incubation for 90 min at room temperature in the dark. The absorbance was measured at 765 nm, and TPC contents were expressed as equivalents of gallic acid, used as the standard (mg eq GA g^-1^ DW).

TF contents in the samples were determined by nitration of the catechol group with sodium nitrate (NaNO_2_) followed by a reaction with aluminium chloride (AlCl_3_) under alkaline conditions (Zhishen et al., 1999). The absorbance was measured at 510 nm, and TF content was expressed in equivalents of catechin, used as the standard (mg eq C. g^−1^ DW).

### 2.10 Statistical analysis

The effects of two experimental factors, biostimulant (with two levels) and water salinity (with four levels), on multiple traits of interest were investigated. The eight treatments resulting from the cross-combination of the two factors were subjected to statistical analyses. Firstly, a two-way analysis of variance (ANOVA) was conducted to evaluate the effects of the biostimulant application, of the salt stress treatments and their interactions. Subsequently, a Tukey’s honestly significant difference (HSD) test was performed at a significance level of p ≤ 0.05 to identify significant differences between treatments when interactions were significant.

To further examine the principal effects of the experimental factors on the analysed traits and extract meaningful insights, a PCA (principal component analysis) was carried out. In this analysis, 27 morphological and biochemical traits were included as active variables, whereas the biostimulant and water salinity were considered supplementary categorical variables that were not involved in the computation of the PCs. Furthermore, the relationship between the traits was evaluated using correlation analysis. Precisely, pairwise Pearson’s correlation coefficients were calculated, and their significance was tested at a level of α = 0.05.

The statistical analyses were carried out with the RStudio software (version 4.1.3), and the following packages: car (Fox and Weisberg, 2019) and emmeans (Lenth, 2023) for ANOVA and *post-hoc* testing; FactoMineR (Le et al., 2008) and corrr (Kuhn et al., 2022) for PCA and correlation analysis, and ggplot2 (Wickham et al., 2016), ggpubr (Kassambara, 2023) and corrplot (Wei and Simko, 2021.) for generating the charts.

## 3 Results

### 3.1 Substrate’s electric conductivity

**Figure 2:**
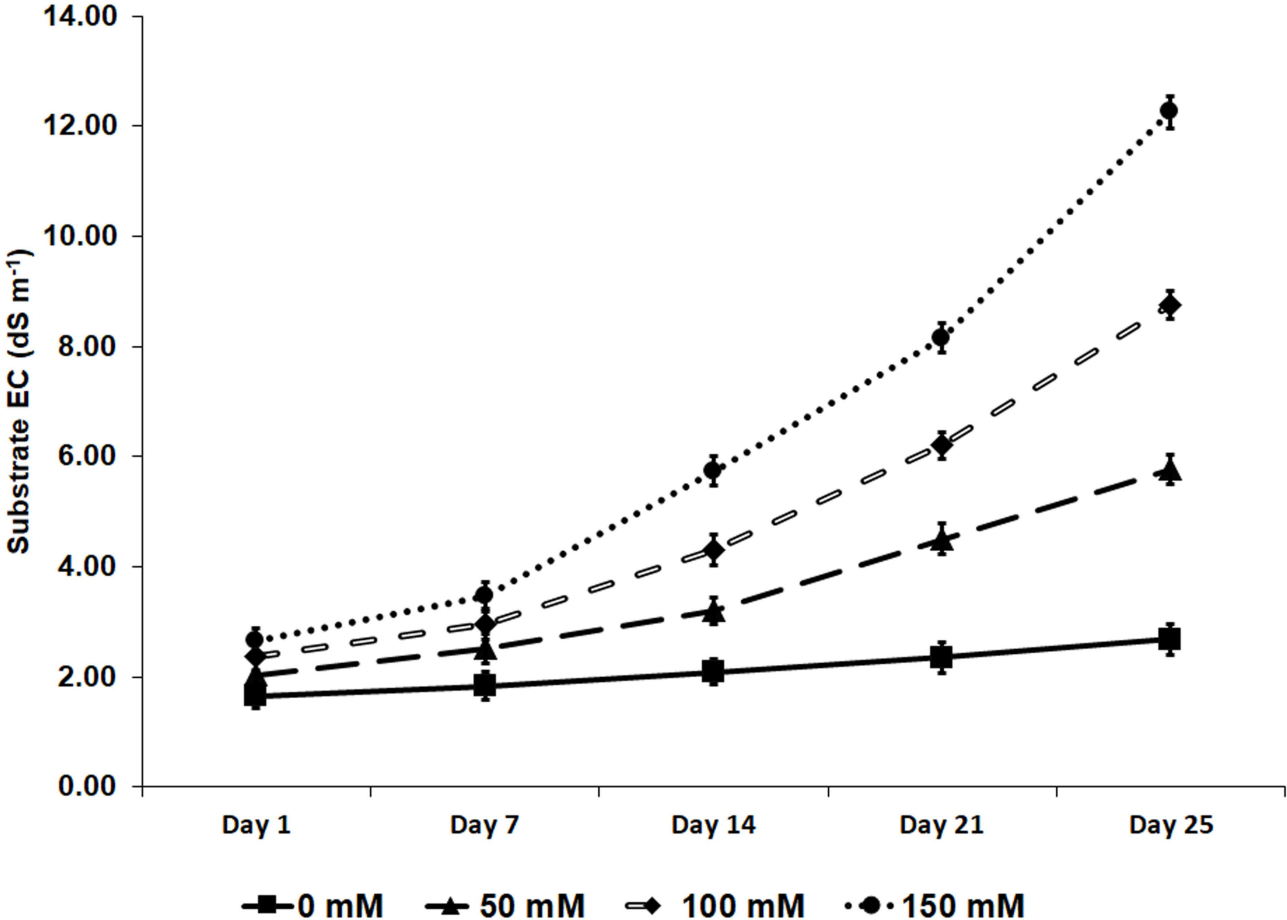
Substrate’s electrical conductivity (EC), measured weekly with a WET sensor during 25 days of salt stress treatments. Values are means of five control pots (no biostimulant added) per treatment ± SE (*n* = 5).

The experiments were stopped 25 days after starting the salt treatments. In response to the time-dependent salt accumulation, the EC in the substrate reached maximum values of 12.3 dS m^-1^ for the treatments with 150 mM NaCl, followed by 8.8 dS m^-1^ and 5.8 dS m^-1^ in the pots irrigated with 100 mM and 50 mM NaCl, respectively. In the absence of salt, the mean EC registered was 2.7 dS m^-1^ (**Figure 2**).

### 3.2 Growth parameters

**Figure 3:**
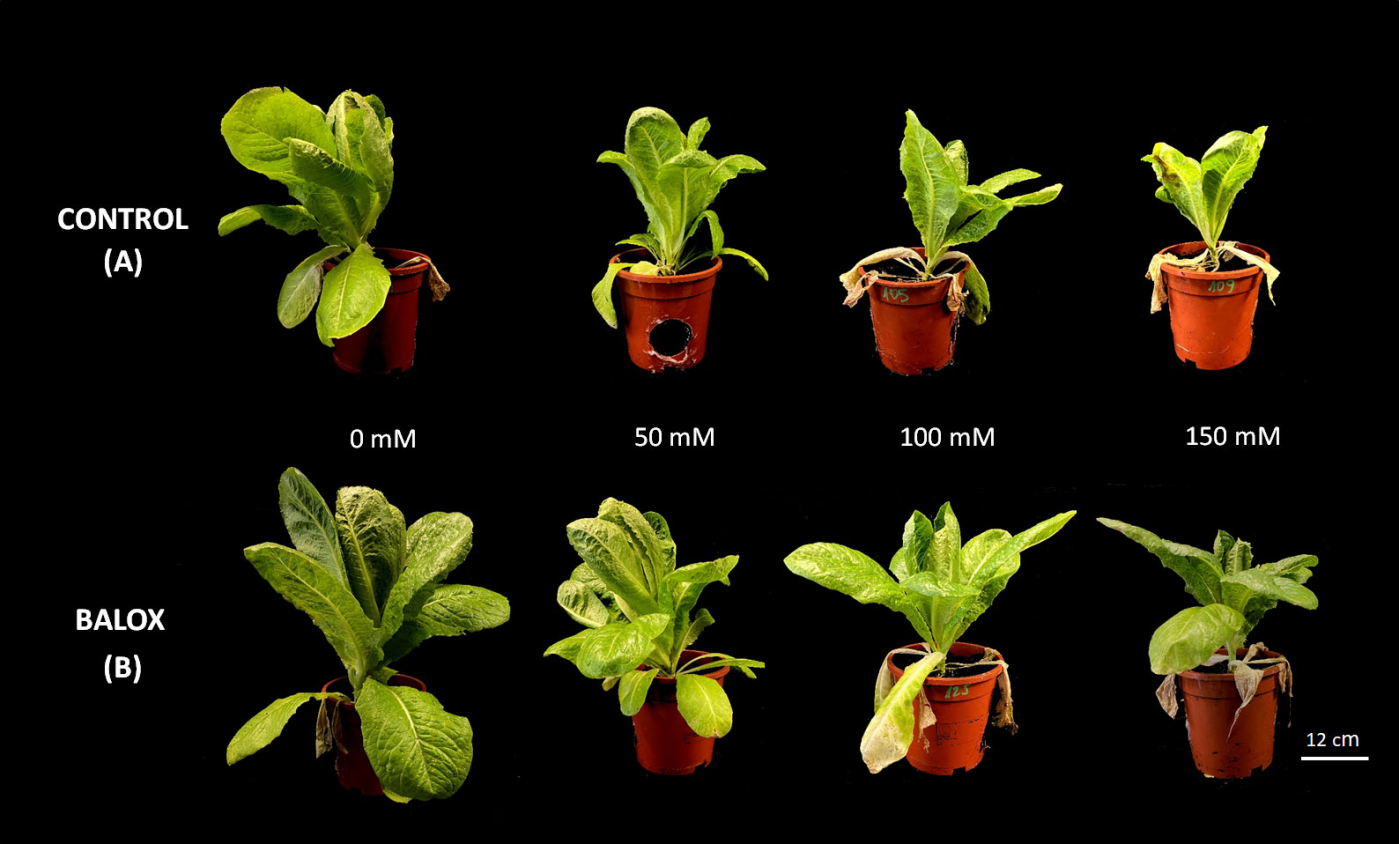
Lettuce plants **(A)** Control, **(B)** treated with BALOX^®^, evaluated after 25 days of treatment with the indicated NaCl concentrations. Dosage of the biostimulant: 0.6 mL L^-1^ of irrigation water.

**Figure 4:**
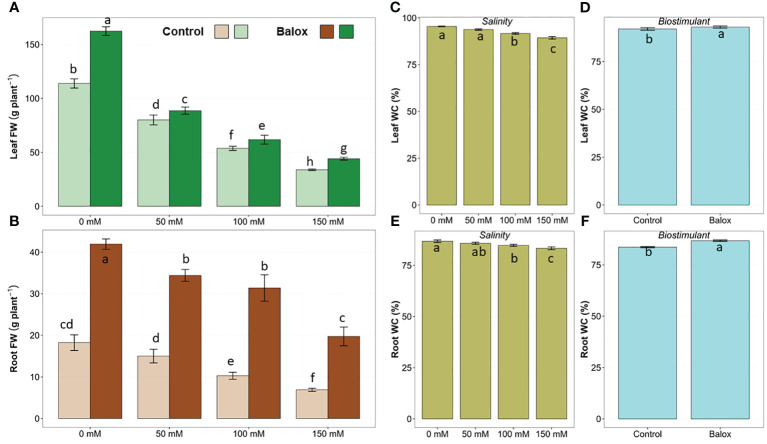
Fresh weight (FW) of leaves **(A)** and roots **(B)** of lettuce plants treated with biostimulant (BALOX^®^) at a dose of 0.6 mL L^-1^ of irrigation water, and control plants without biostimulant. Treatment time: 25 days; salinity levels: 0 mM, 50 mM, 100 mM, 150 mM NaCl. Values are means ± SE (*n* = 5). Effect on water content of leaves and roots of salinity **(C, E)** and the biostimulant **(D, F)**. Different letters above the bars indicate significant differences between treatments, according to Tukey’s test (*p* ≤ 0.05).

The two-way ANOVA indicated significant effects of salinity level and biostimulant application for all evaluated growth parameters (**Table 1**). Their interactions were significant only for the fresh weight of roots and leaves but not for their water content. Salt stress inhibited the growth of romaine lettuce plants in a concentration-dependent manner, both in the absence and the presence of the biostimulant (**Figure 3**). Regarding the analysed parameters, the fresh weight (FW) of roots and leaves showed significant decreases. In those treatments without biostimulant, a reduction of more than 62% in root FW (**Figure 4A**) and more than 70% in leaf FW (**Figure 4B**) was found in plants subjected to 150 mM NaCl, compared to the plants grown without salt. In the presence of the biostimulant, the corresponding reduction percentages amounted to 53% and 73% for root and leaf FW, respectively (**Figures 4A, B**). In addition, a slight but statistically significant water loss was observed in roots and leaves in response to the salt treatments (**Figures 4C, E**).

**Table 1:** Two-way ANOVA (p values) considering the effect of biostimulant (B), salinity (S) and their interactions (B x S) on plant growth parameters of lettuce plants (FWr, fresh weight of roots; FWl, fresh weight of leaves; WCr, water content of roots; WCl, water content of leaves) and photosynthetic pigments (tChl, total chlorophylls; Caro, carotenoids).

	df	FWr	FWl	WCr	WCl	tChl	Caro
**Biostimulant (B)**	1	.0000 ***	.0000 ***	.0000 ***	.0000 ***	.0000 ***	.0000 ***
**Salinity (S)**	3	.0000 ***	.0000 ***	.0000 ***	.0000 ***	.0000 ***	.0000 ***
**B x S**	3	.0321 *	.0000 ***	.6498 ns	.8515 ns	.0000 ***	.0410 *

*, *** significant at p = 0.05, and 0.001, respectively; ns, not significant.

The addition of the biostimulant stimulated plant growth significantly in all treatments, with and without salt. Root FW increased ~2.3-fold at 0 mM and 50 mM NaCl and almost 3-fold at higher salt concentrations (100 mM and 150 mM NaCl) (**Figure 4A**). Likewise, leaf FW increased 1.4-fold in non-saline conditions and 1.3-fold in the presence of 150 mM NaCl (**Figure 4A**). Applying the biostimulant also improved the plants’ hydration level, as small but significant increases in the water content of leaves (**Figure 4D**) and roots (**Figure 4F**) were detected.

### 3.3 Root evaluation

**Figure 5:**
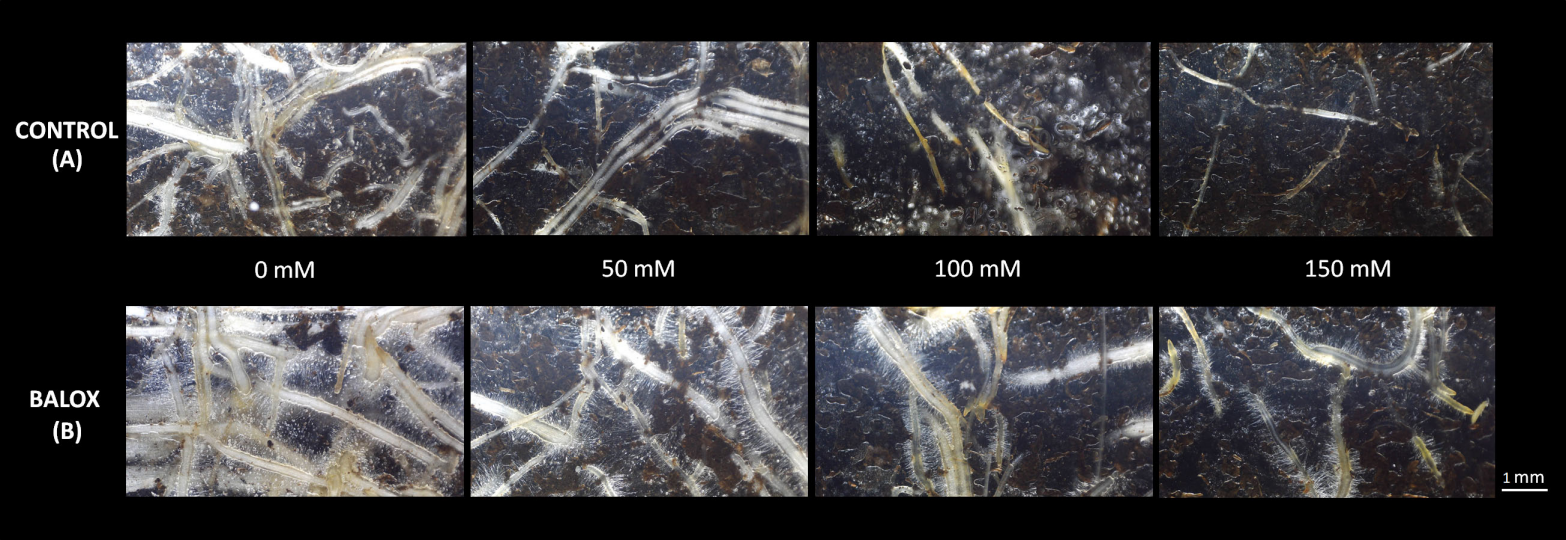
Lettuce plant roots **(A)** Control, **(B)** treated with BALOX^®^, evaluated after 25 days of treatment with NaCl concentrations. Dosage of biostimulant: 0.6 mL L^-1^ of irrigation water. Photos were taken with a minirhizotron camera, model ST 21.

The analysis of variance indicated a significant effect of the biostimulant, salinity and their interactions for all root evaluations (**Table 2**). As shown in **Figure 5**, increasing root damage caused by salinity was observed in parallel with the increase of the NaCl concentration in the irrigation water. Salt inhibited root growth and caused root burn. However, the damage caused by salinity was less pronounced in the plants treated with the biostimulant. The biostimulant also showed a protective effect on roots and absorbing hairs, even under high salinity conditions (**Figure 5**).

**Table 2:** Two-way ANOVA (p values) considering the effect of biostimulant (B), salinity (S) and their interactions (B x S) on the different categories of root area of lettuce plants in the plane projected with the minirhizotron camera.

	df	RA	< 0.25 mm	0.25 - 0.50 mm	0.50 - 2.0 mm	2.0 - 5.0 mm	> 5.0 mm
**Biostimulant (B)**	1	.0000 ***	.0000 ***	.0000 ***	.0000 ***	.0000 ***	.0000 ***
**Salinity (S)**	3	.0000 ***	.0000 ***	.0000 ***	.0000 ***	.0000 ***	.0000 ***
**B x S**	3	.0000 ***	.0003 ***	.0145 *	.0000 ***	.0023 **	.0000 ***

*, **, *** significant at p = 0.05, 0.01 and 0.001, respectively; ns, not significant.

**Figure 6:**
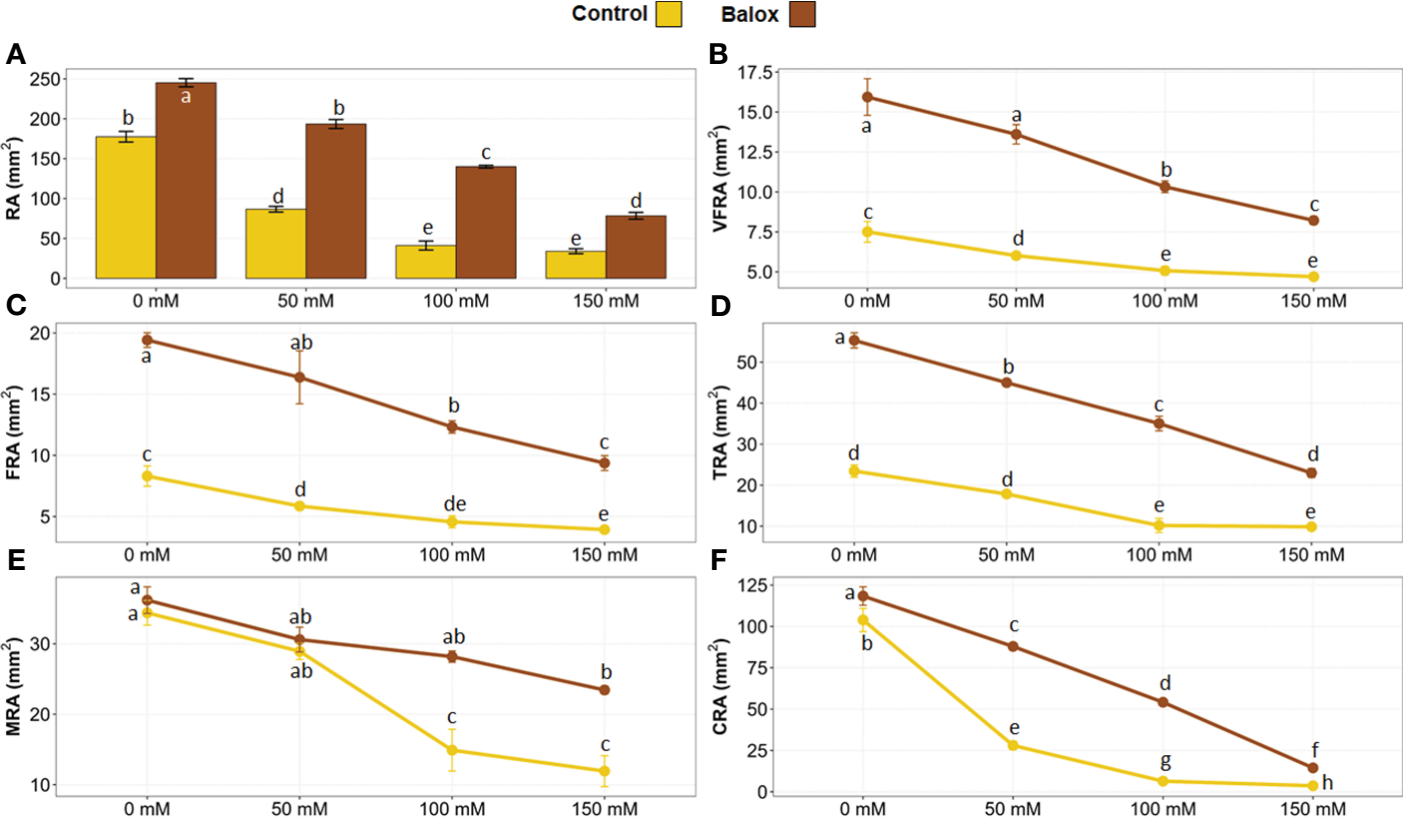
Root area of lettuce plants (RA) **(A)** in the plane projected with the minirhizotron camera model ST 21 during the real-time evaluation after 25 days under salt stress. The WinRHIZO (WRh) programme allowed the areas to be classified into five categories according to root diameter:< 0.25 mm, area of very fine roots (VFRA) **(B)**; from 0.25 to 0.50 mm, area of fine roots (FRA) **(C)**; from 0.50 to 2.0 mm, thin root area (TRA) **(D)**; from 2.0 to 5.0 mm, medium root area (MRA) **(E)**; and > 5.0 mm, coarse root area (CRA) **(F)**. The plants were grown at four salinity levels (0 mM, 50 mM, 100 mM, 150 mM NaCl), treated or not (Control) with the biostimulant (BALOX^®^) at a dose of 0.6 mL L^-1^ of irrigation water. Values are means ± SE (*n* = 5). Different letters above the bars indicate significant differences between treatments, according to Tukey’s test (*p* ≤ 0.05).

Root evaluations were performed in real-time (without removing the plants from the pots) at the end of the treatments. For this, a minirhizotron camera was connected to the computer and inserted inside the pots through the transparent tubes installed before transplanting (**Figure 1D**). Using the camera, root images were obtained in a rectangular plane (10 x 8 mm) from the camera lens projected onto the substrate (**Figure 1D**). The images were then analysed with the WinRHIZO software to calculate the area occupied by the roots on this projected plane (**Figure 6A**). The results showed that salt stress caused a significant inhibition of root growth and the reduction of root surface area in the space evaluated with the minirhizotron camera in real-time (80 mm^2^), in parallel to the increase of the NaCl concentration (**Figure 6A**). For instance, a reduction of 81% in the mean root area (34.1 mm^2^) was observed in plants treated with 150 mM NaCl, compared to the non-stressed plants, which had a mean root area of 177.5 mm^2^ (**Figure 6A**). BALOX^®^ application enhanced root growth and induced a significant increase in root area under all experimental conditions tested, especially in the presence of salt, where the most significant differences were detected. For example, increases of 2.2- and 3.4-fold were observed in the root area of BALOX^®^-treated plants in the presence of 50 mM and 100 mM NaCl, respectively, compared to the control plants not treated with the biostimulant (**Figure 6A**).

WinRHIZO made it possible to measure the total area of the root in the photograph and segment that area by categories or ranges of interest according to root diameter. In the present work, five categories of agronomic interest have been established, namely, very fine roots (< 0.25 mm), fine roots (0.25 to 0.50 mm), thin roots (0.5 to 2.0 mm), medium roots (2.0 to 5.0 mm) and coarse roots (> 5.0 mm). The area corresponding to each root category decreased progressively with increasing salt concentrations in the presence and the absence of BALOX^®^. For all root categories and treatments, the root area of biostimulant-treated plants was larger than that of the control plants, as shown in **Figures 6B–F**. The diameter of the roots is associated with their degree of absorption; the smaller the diameter, the greater the absorption, as root hairs are predominantly located in the thinnest roots. Conversely, the larger the root diameter, the lower the absorption since thick roots are primarily responsible for the fixation and support of the plant in the substrate, and not so much for nutrient uptake from the soil.

These results also indicated that high concentrations of NaCl caused the loss of absorbent hairs and, therefore, reduced the absorption area, an effect more pronounced in control plants not treated with BALOX^®^. For example, **Figure 6B** shows that, in the absence of the biostimulant, the minimum mean value of the area of very fine roots was 4.70 mm^2^, measured in the presence of 150 mM NaCl. However, under the same conditions, the plants treated with the biostimulant doubled the root area compared to the control, also presenting a greater absorption area.

### 3.4 Quantification of photosynthetic pigments

**Figure 7:**
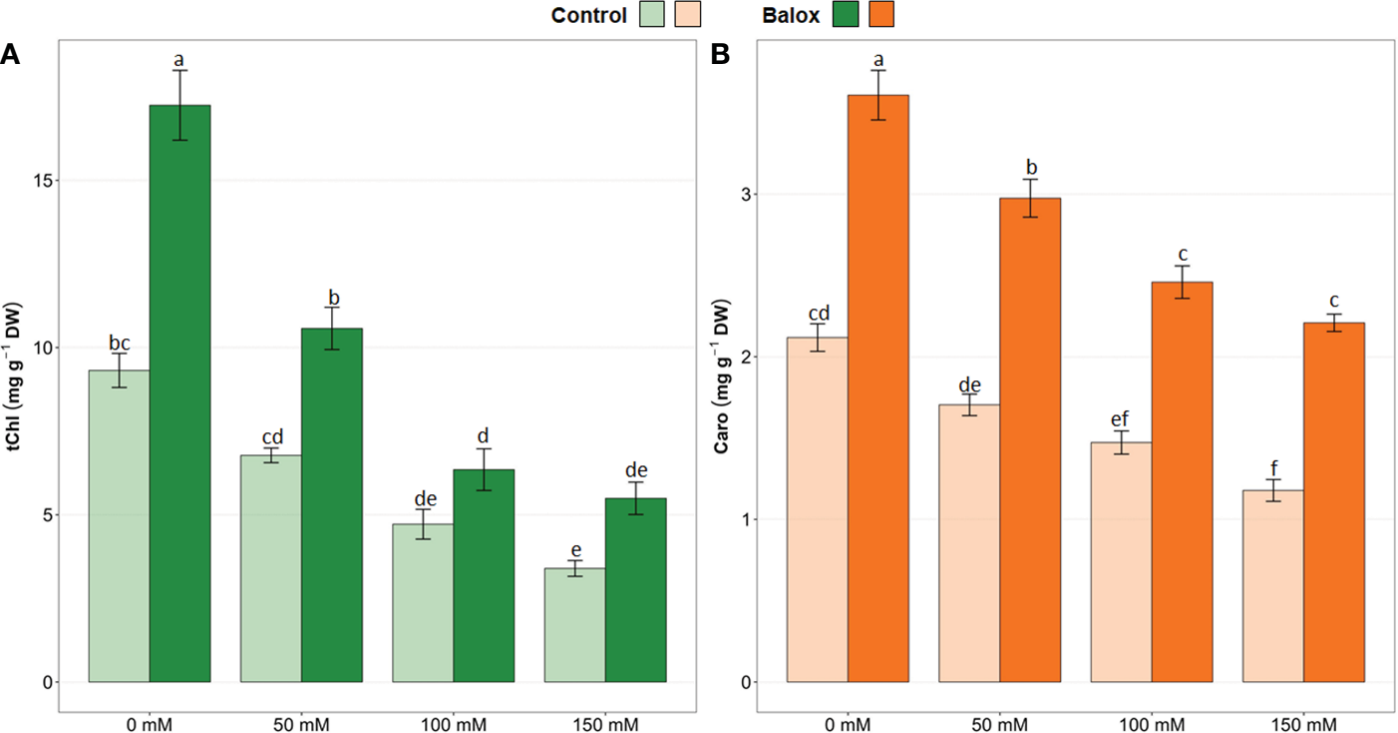
Total chlorophyll (tChl), **(A)**, and total carotenoids (Caro), **(B)** contents in lettuce plants treated or not (Control) with biostimulant (BALOX^®^) at a dose of 0.6 mL L^-1^ of irrigation water, at four salinity levels (0 mM, 50 mM, 100 mM, 150 mM NaCl). Values are means ± SE (*n* = 5). Different letters above the bars indicate significant differences between treatments, according to Tukey’s test (*p* ≤ 0.05).

The two-way ANOVA detected statistical significance for the application of biostimulant (B), the level of salinity (S) and their interactions (B x S), as shown in **Table 1**. The effect on lettuce plants of increasing salinity levels may be related to the decrease in total chlorophylls (tChl) and carotenoids (Caro). In fact, a significant decrease in the levels of tChl (**Figure 7A**) and Caro (**Figure 7B**) was observed in all salt stress treatments compared to the plants not subjected to stress (0 mM NaCl). Plants exposed to high concentrations of NaCl (100 and 150 mM) were severely affected, exhibiting a decrease of 64% and 40% in tChl and Caro levels, respectively. However, applying the biostimulant at the base of the plant caused tChl and Caro leaf concentrations to increase significantly in all treatments compared to the controls. For example, in plants grown in the absence of salt, the use of BALOX^®^ increased tChl content by 1.8-fold and Caro by 1.6-fold. On the other hand, in plants subjected to 150 mM NaCl, BALOX^®^ application resulted in increases of tChl and Caro of 1.6- and 1.9-fold, respectively, with respect to the controls without biostimulant (**Figure 7**).

### 3.5 Quantification of ions

**Figure 8:**
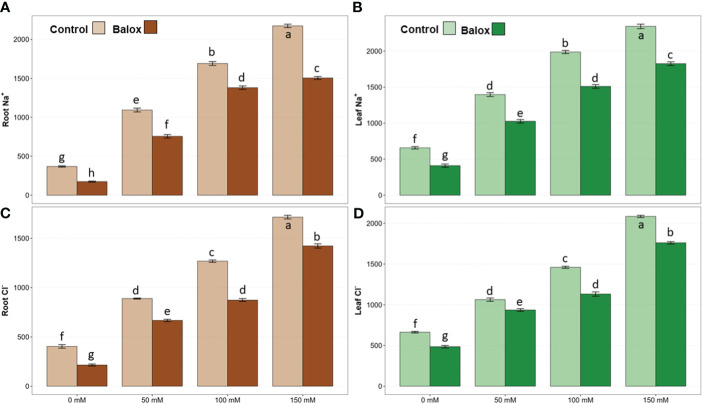
Concentration in roots (left panels) and leaves (right panels) of sodium (Na^+^) **(A, B)** and chloride (Cl^-^) **(C, D)** in lettuce plants treated or not (Control) with biostimulant (BALOX^®^) at a dose of 0.6 mL L^-1^ of irrigation water, for 25 days at four salinity levels (0 mM, 50 mM, 100 mM, 150 mM NaCl). Values are means ± SE (*n* = 5). Different letters above the bars indicate significant differences between treatments, according to Tukey’s test (*p* ≤ 0.05).

The concentration of monovalent (Na^+^, Cl^-^, K^+^) and divalent (Ca^2+^) ions was determined in the roots and leaves of *L. sativa* plants after 25 days of treatment with increasing NaCl concentrations. The analysis of variance revealed significant effects of salinity and biostimulant for all analysed ions, in roots and leaves. The interactions of the two factors were significant, except those of K^+^ in roots and leaves and Ca^2+^ in leaves (**Table 3**). A significant increase in the content of Na^+^ and Cl^-^ ions in roots and leaves was observed in parallel to the increase in external salinity, with and without the biostimulant (**Figures 8A–D**). For example, in the absence of BALOX^®^ and under severe salt stress conditions (150 mM NaCl), maximum (and similar) average contents of 2173 and 2344 µmol g^-1^ DW were determined for Na^+^ in roots and leaves, respectively (**Figures 8A, B**); these values represent increases of 5.9-fold in roots and ca. 3.6-fold in leaves, with respect to the plants grown without salt. Similar variation patterns were observed for Cl^-^ contents, reaching 1715 µmol g^-1^ DW in roots in the presence of 150 mM NaCl, a 4.2-fold increase over the value in non-stressed plants (**Figure 8C**), and 2085 µmol g^-1^ DW in leaves, a 3.1-fold increase, approximately (**Figure 8D**).

**Table 3:** Two-way ANOVA (p values) considering the effect of biostimulant (B), salinity (S) and their interactions (B x S) on ion contents in roots (r) and leaves (l) of lettuce plants.

	df	Na(r)	Cl(r)	K(r)	Ca(r)	Na(l)	Cl(l)	K(l)	Ca(l)
**Biostimulant (B)**	1	.0000 ***	.0000 ***	.0000 ***	.0000 ***	.0000 ***	.0000 ***	.0006 ***	.0000 ***
**Salinity (S)**	3	.0000 ***	.0000 ***	.0000 ***	.0000 ***	.0000 ***	.0000 ***	.0000 ***	.0000 ***
**B x S**	3	.0000 ***	.0000 ***	.0883 ns	.0007 ***	.0000 ***	.0000 ***	.5243 ns	.6747 ns

*** significant at p = 0.001; ns, not significant.

Application of the biostimulant led to a significant reduction in root and leaf contents of both toxic ions, Na^+^ and Cl^-^, under all experimental conditions tested (**Figures 8A–D**). For example, in the absence of salt, Na^+^ concentrations decreased by 53% in roots (**Figure 8A**) and 38% in leaves (**Figure 8B**) in response to the biostimulant. At the highest NaCl concentration tested, 150 mM, the corresponding reduction percentages were 31% in roots and 22% in leaves (**Figures 8A, B**). Similar to Na^+^, BALOX^®^-induced relative reductions in Cl^-^ concentrations were more pronounced in roots than leaves and in non-stressed than salt-treated plants (**Figures 8C, D**).

**Figure 9:**
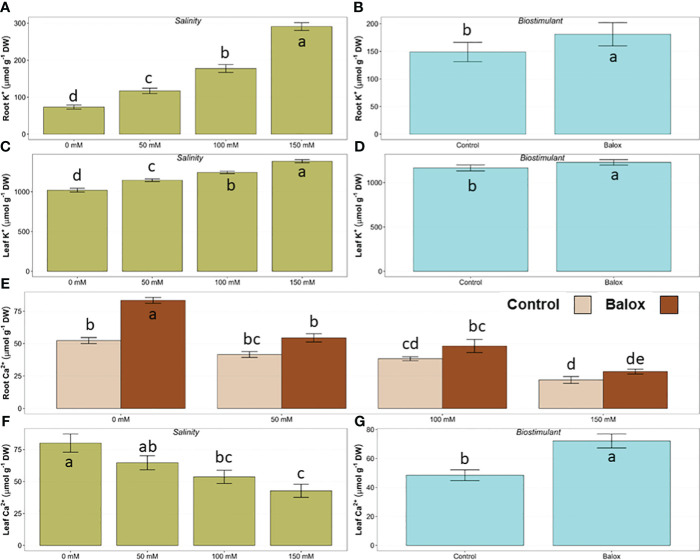
Effect of salinity **(A, C, F)** and the biostimulant **(B, D, G)** on the concentration of potassium (K^+^) ions in roots **(A, B)** and leaves **(C, D)** and the concentration of calcium (Ca^2+^) ions in leaves **(F, G)** in lettuce plants treated for 25 days at four salinity levels (0 mM, 50 mM, 100 mM, 150 mM NaCl). The significant interaction of the factors (salinity and biostimulant) for Ca^2+^ in roots is shown in panel **(E)** for plants treated or not (Control) with biostimulant (BALOX^®^) at a dose of 0.6 mL L^-1^ of irrigation water. Different letters above the bars indicate significant differences between treatments, according to Tukey’s test (*p* ≤ 0.05).

Regarding the ‘physiological’ cation, K^+^, its concentrations also increased in roots and leaves in the absence of the biostimulant, in parallel to the increase in substrate salinity; the relative rise in K^+^ contents was more pronounced in roots (about 3.9-fold higher in the presence of 150 mM NaCl than in the absence of salt) than in leaves (1.4-fold higher) (**Figures 9D, F**). The application of the biostimulant resulted in a slight increase of mean K^+^ contents under all conditions tested, and the effect of the biostimulant detected by the two-way ANOVA was significant for roots (**Figure 9E**) and leaves (**Figure 9G**). It should also be noticed that, for all treatments, K^+^ concentrations were substantially higher in leaves than in roots, but such large differences were not observed for the other analysed ions (**Figures 8**, **9**).

Contrary to the variation patterns of monovalent ions, the salt treatments resulted in a progressive, significant reduction of Ca^2+^ levels in the roots and leaves of the lettuce plants. For example, in control plants (no biostimulant added), watering with 150 mM NaCl reduced Ca^2+^ concentrations by about 60% and 50% in roots and leaves, respectively. Biostimulant application increased the average values of Ca^2+^ contents for both plant organs and all treatments, between 1.3 and 1.7-fold. The analysis of variance (**Table 3**) indicated a significant effect of the interaction of the two factors (biostimulant and salinity) only for roots, with maximum values in control plants treated with BALOX^®^ (**Figure 9A**). Regarding calcium concentrations in leaves, although both the salinity treatment (**Figure 9B**) and the biostimulant (**Figure 9C**) showed significant effects, decreasing in the former and increasing in the latter, their interaction was not significant.

### 3.6 Quantification of osmolytes

**Figure 10:**
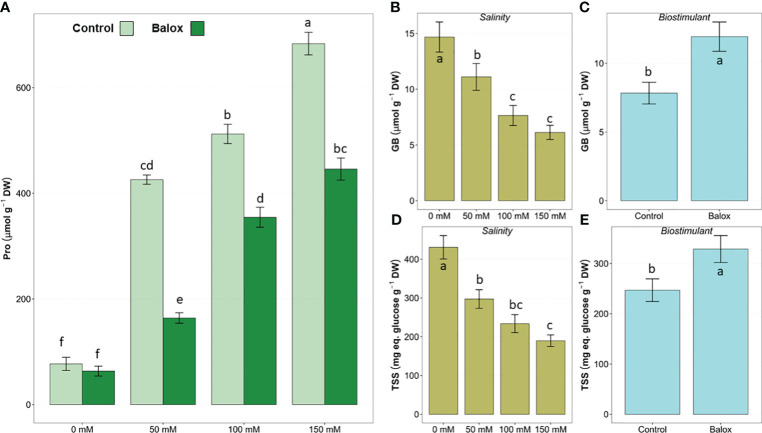
Leaf proline (Pro) **(A)** contents in lettuce plants treated or not (Control) for 25 days with biostimulant (BALOX^®^) at a dose of 0.6 mL L^-1^ of irrigation water at four salinity levels (0 mM, 50 mM, 100 mM, 150 mM NaCl). Values for proline are means ± SE (*n* = 5). Effects of salinity **(B, D)** and the biostimulant **(C, E)** are indicated for GB **(B, C)** and TSS **(D, E)**. Different letters above the bars indicate significant differences between treatments, according to Tukey’s test (*p* ≤ 0.05).

The two-way ANOVA performed for osmolyte concentrations (**Table 4**) revealed a significant effect of treatment and biostimulant for the three osmolytes analysed, but the interaction of the two factors was significant only for proline (**Figure 10**). Leaf proline (Pro) content increased significantly in response to increasing NaCl concentration in the stress treatments, reaching levels approximately 9-fold higher at 150 mM NaCl than in non-saline conditions (**Figure 10A**).In contrast to Pro, the levels of glycine betaine (GB) (**Figure 10B**) and total soluble sugars (TSS) (**Figure 10D**) gradually decreased as the salinity increased.

**Table 4:** Two-way ANOVA (p values) considering the effect of biostimulant (B), salinity (S) and their interactions (B x S) on concentrations of osmolytes (Pro, proline; GB, glycine betaine; TSS, total soluble sugars), oxidative stress markers (MDA, malondialdehyde; H_2_O_2_, hydrogen peroxide), and antioxidants (TPC, total phenolic compounds; TF, total flavonoids).

	df	Pro	GB	TSS	MDA	H_2_O_2_	TPC	TF
Biostimulant (B)	**1**	.0000 ***	.0000 ***	.0003 ***	.0000 ***	.0000 ***	.0002 ***	.0925 ns
Salinity (S)	**3**	.0000 ***	.0000 ***	.0000 ***	.0000 ***	.0000 ***	.1056 ns	.0001 ***
BxS	**3**	.0000 ***	.1443 ns	.7020 ns	.0002 ***	.0169 *	.9490 ns	.9911 ns

*, *** significant at p = 0.05, and 0.001, respectively; ns, not significant.

In the presence of the biostimulant, Pro concentrations decreased significantly in all salt-treated plants compared to those subjected to the same salt concentration without the biostimulant; for example, in the presence of 50 mM NaCl, Pro contents were reduced by 62%, and by 35% in the 150 mM NaCl treatment. However, no significant differences were observed in the absence of salt (**Figure 10A**). Application of the biostimulant led to a significant increase in GB (**Figure 10C**) and TSS (**Figure 10E**) contents.

### 3.7 Determination of oxidative stress levels

**Figure 11:**
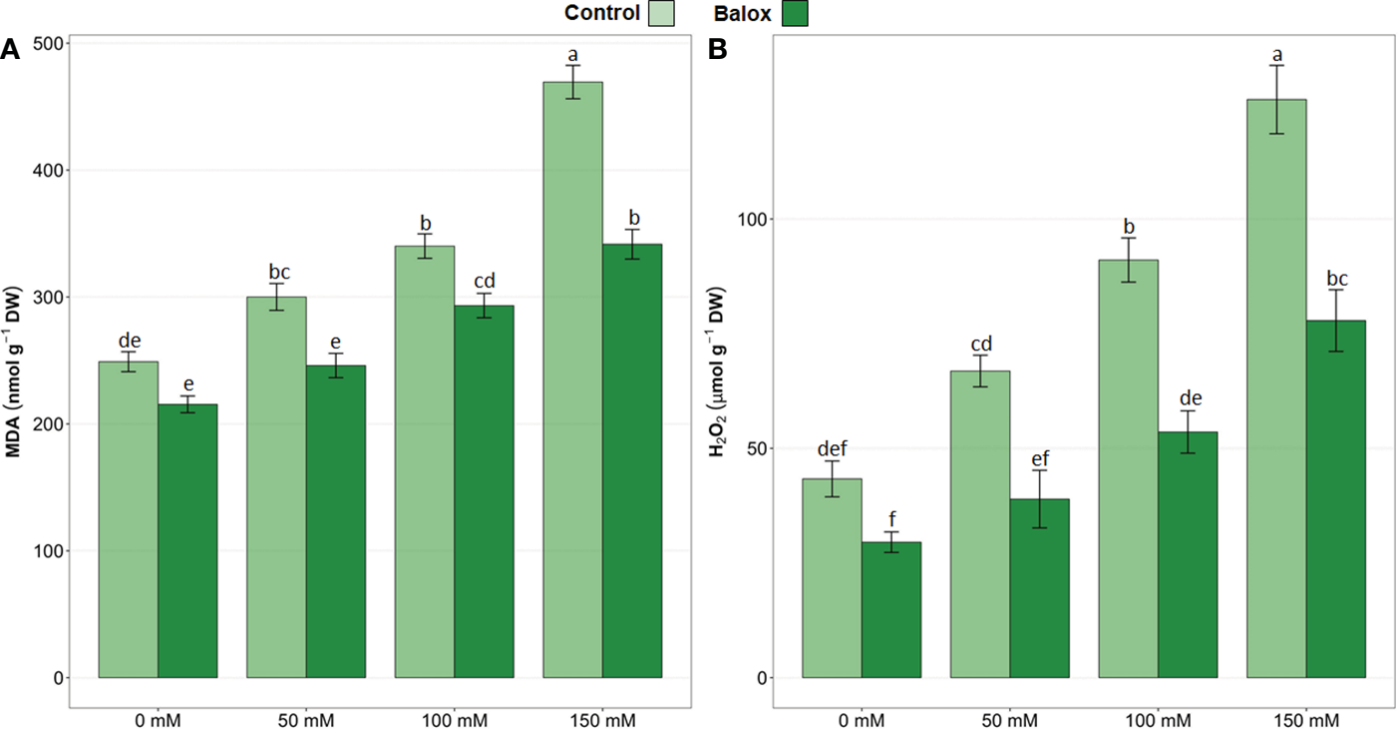
Malondialdehyde (MDA) **(A)**, and hydrogen peroxide (H_2_O_2_) **(B)** contents in leaves of lettuce plants treated or not (Control) for 25 days with biostimulant (BALOX^®^) at a dose of 0.6 mL L^-1^ of irrigation water with four salinity levels (0 mM, 50 mM, 100 mM, 150 mM. Values are means ± SE (*n* = 5). Different letters above the bars indicate significant differences between treatments, according to Tukey’s test (*p* ≤ 0.05).

The results of the two-way ANOVA in **Table 4** indicate statistical significance for the two factors (salinity and biostimulant) and their interaction. Malondialdehyde (MDA) concentrations increased significantly and progressively with increasing external salinity, reaching the highest value (469 nmol g^-1^ DW) in plants grown without the biostimulant under severe salinity conditions (150 mM NaCl); this represented an increase of about 1.9-fold over the non-stressed plants (**Figure 11A**). Similarly, hydrogen peroxide (H_2_O_2_) levels also increased substantially in response to the salt treatments, up to 126.05 µmol g^-1^ DW at 150 mM NaCl, about 3-fold higher than under non-saline conditions (**Figure 11B**).

The application of BALOX^®^ significantly reduced the levels of MDA and H_2_O_2_ in the plants subjected to all salt treatments, whereas the reductions observed for both oxidative stress biomarkers in the absence of salt were not statistically significant (**Figure 11**). For example, in the presence of 150 mM NaCl, MDA and H_2_O_2_ contents decreased by 27% and 38%, respectively, compared to the plants not treated with the biostimulant (**Figure 11**).

### 3.8 Quantification of antioxidant compounds

**Figure 12:**
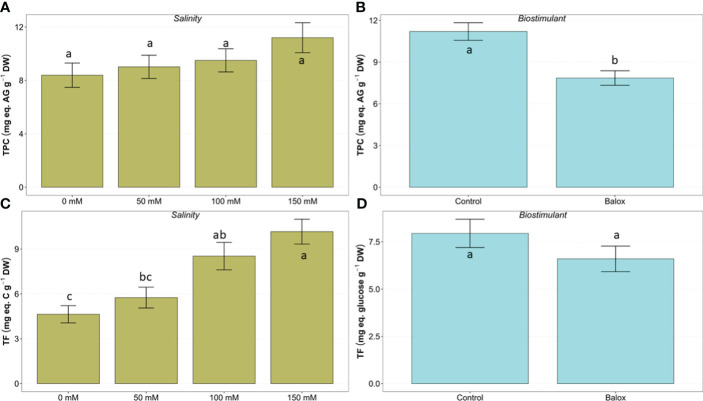
Effect of salinity **(A, C)** and the biostimulant **(B, D)** on the leaf content of total phenolic compounds (TPC) **(A, B)**, and total flavonoids (TF) **(C, D)** in lettuce plants treated for 25 days at four salinity levels (0 mM, 50 mM, 100 mM, 150 mM. Different letters above the bars indicate significant differences between treatments, according to Tukey’s test (*p* ≤ 0.05).

As expected, oxidative stress due to salinity conditions appeared to induce the synthesis of representative antioxidant compounds in lettuce plants, such as total phenolic compounds (TPC) and total flavonoids (TF). The mean contents of these metabolites increased progressively, in parallel to the NaCl concentration in the irrigation solution (**Figure 12**). However, despite the clear increasing trend, these differences were significant only for TF (**Figure 12C**) but not for TPC (**Figure 12A**), in agreement with the two-way ANOVA results (**Table 4**).

The application of BALOX^®^ reduced the average TPC and TF but the differences observed between plants treated and not treated with the biostimulant were statistically significant only for the former (**Figure 12B**) but not for the latter (**Figure 12D**).

### 3.9 Correlation matrix and relative expressions

**Figure 13:**
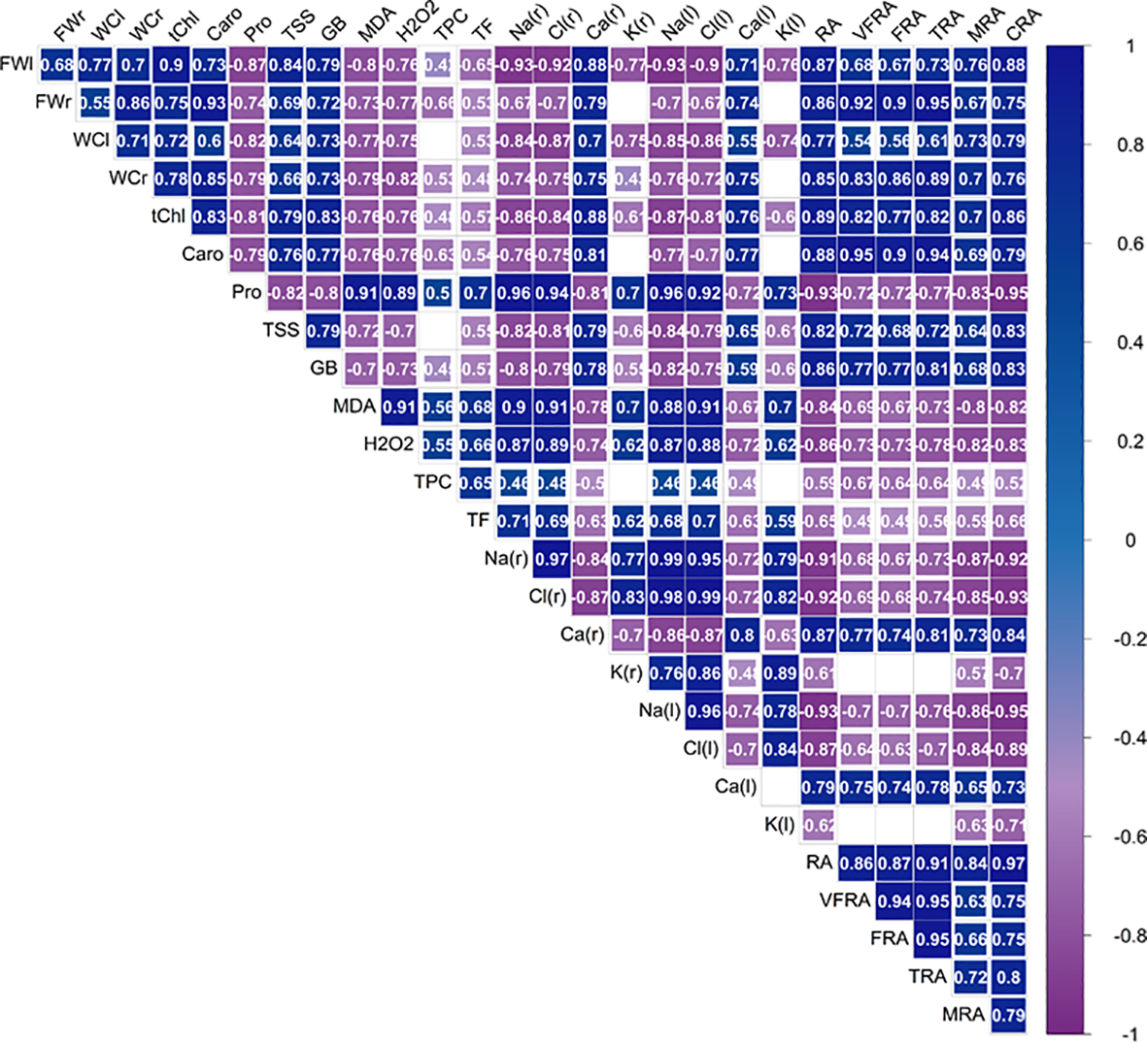
Pearson correlation analysis for the effect of the four salinity levels (0 mM, 50 mM, 100 mM, 150 mM NaCl), control and biostimulant on biochemical and growth traits. leaf fresh weight (FWl), root fresh weight (FWr), leaf water content (WCl), root water content (WCr), total chlorophyll (tChl), total carotenoids (Caro), proline (Pro), total soluble sugars (TSS), glycine betaine (GB), malondialdehyde (MDA), hydrogen peroxide (H_2_O_2_), total phenolic compounds (TPC), total flavonoids (TF), Na^+^ concentration in roots [Na (r)] and leaves [Na (l)], Cl^-^ concentration in roots [Cl (r)] and leaves [Cl (l)], K^+^ concentration in roots [K (r)] and leaves[K (l)], Ca^2+^ concentration in roots [Ca (r)] and leaves [Ca (l)], area of roots in the plane projected with the minirhizotron camera (RA), area of very fine roots (VFRA), area of fine roots (FRA), area of thin roots (TRA), area of medium roots (MRA), area of coarse roots (CRA).

A correlation matrix was performed to investigate the interrelationships and multicollinearity amongst the variables and to provide valuable insights into potential causal relationships (**Figure 13**). Upon observing the correlation matrix, it became apparent that biometric parameters, yield, and pigment contents were inversely correlated with the content of sodium, chloride and potassium ions. These three ions, in turn, were inversely correlated with the calcium content. This indicates that the increased accumulation of sodium and chloride ions under salinity stress did not limit but instead stimulated the accumulation of potassium while reducing calcium levels. The content of Na^+^, Cl^-^ and K^+^ ions was also positively correlated with the proline content but not with the concentrations of sugars and glycine betaine, suggesting that proline is the primary osmoregulator produced by the plant under osmotic stress conditions. Proline, in turn, is positively correlated with the content of MDA and H_2_O_2_, indicating that despite an increase in proline production under salinity conditions, the plant still experienced a higher level of oxidative stress. A similar trend was observed for TPC and the subclass of TF, antioxidant compounds that have been found to be effective in mitigating salinity’s adverse effects in lettuce plants alongside proline.

### 3.10 Multivariate analysis of the results

**Figure 14:**
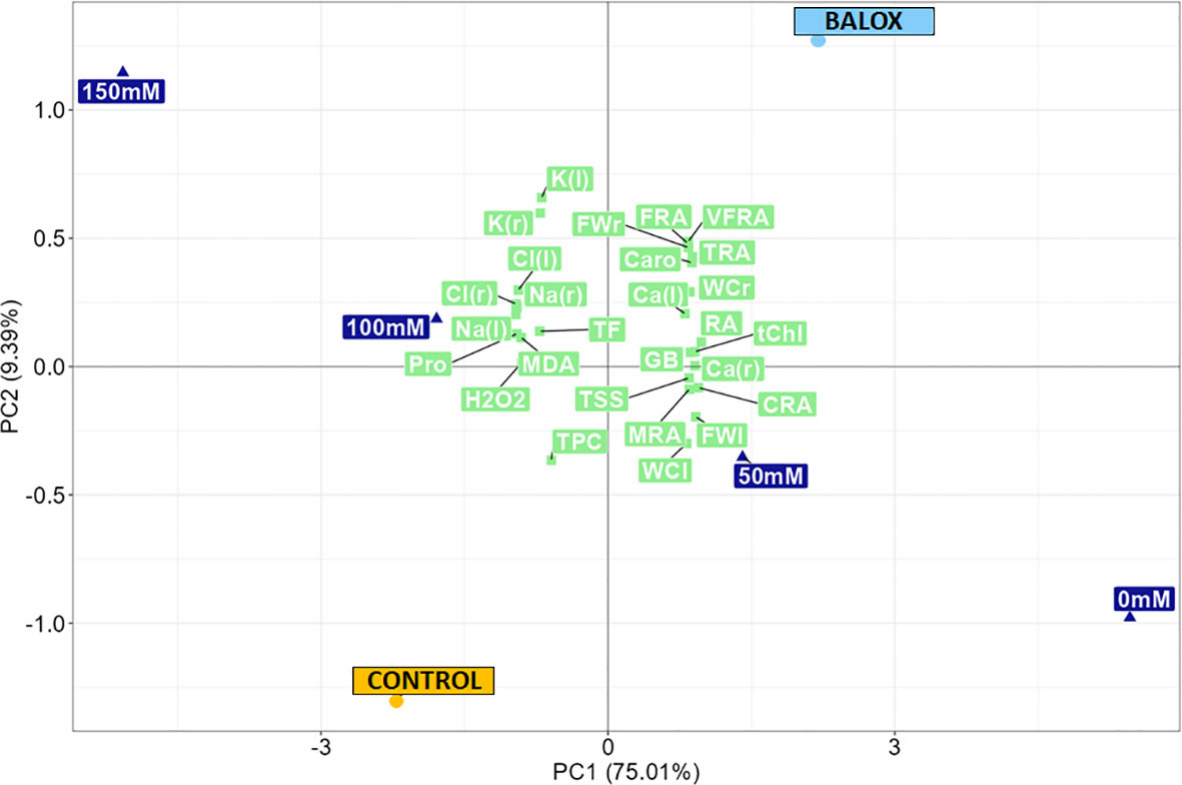
Principal component analysis (PCA) of growth and biochemical parameters in lettuce plants subjected to four salinity conditions (0 mM, 50 mM, 100 mM, 150 mM NaCl), treated or not (Control) with biostimulant (BALOX^®^). The percentages of the total variability are explained by the first and second components (73.28% and 8.72%, respectively). root fresh weight (FWr), leaf fresh weight (FWl), root water content (WCr), leaf water content (WCl), root area in the plane projected with the minirhizotron camera (RA), classified into five categories according to root diameter:< 0.25 mm, [area of very fine roots (VFRA)], from 0.25 to 0.50 mm, [area of fine roots (FRA)], from 0.50 to 2.0 mm, [thin root area (TRA)], from 2.0 to 5.0 mm, [medium root area (MRA)], and > 5.0 mm, [coarse root area (CRA)], total chlorophyll (tChl), total carotenoids (Caro), Na^+^ concentration in roots [Na (r)] and leaves [Na (l)], Cl^-^ concentration in roots [Cl (r)] and leaves [Cl (l)], K^+^ concentration in roots [K (r)] and leaves [K (l))], Ca^2+^ concentration in roots [Ca (r)] and leaves [Ca (l)], proline (Pro), total soluble sugars (TSS), glycine betaine (GB), malondialdehyde (MDA), hydrogen peroxide (H_2_O_2_), total phenolic compounds (TPC), total flavonoids (TF).

To obtain a holistic view of the results, a principal component analysis (PCA) was performed, based on the study of the relationships of the growth and biochemical parameters (**Figure 14**), measured in control *Lactuca sativa* plants and those treated with the biostimulant (BALOX^®^) under salt stress conditions. A percentage of 84.4 of the total variability was covered by the first two components (**Figure 14**). The first principal component (PC1), which accounted for 75.01% of the total variation, provided a clear separation of the effects of the four salinity levels (0 mM, 50 mM, 100 mM, 150 mM NaCl) and allowed distinguishing their relative impact on the two treatments evaluated (Control and BALOX^®^).

The BALOX^®^ barycentre and the lower salt concentration levels, 0 mM (non-saline) and 50 mM NaCl, were located on the positive side of the X-axis, indicating a positive correlation with PC1. In contrast, both the barycentre of the control and the highest salt stress levels (100 mM, 150 mM) were located on the negative side of the X-axis, revealing a negative correlation with PC1. This arrangement confirmed that the use of BALOX^®^ influenced lettuce plants under salt stress conditions (both lower and higher NaCl concentrations) to behave similarly to unstressed or less stressed plants, whereas it was evident that control plants (without the biostimulant) appeared to be more severely affected by salt stress.

The second axis explained 9.39% of the total variation, distinguishing between the two treatments (Control and BALOX^®^) regarding growth and biochemical parameters. The control was associated with higher levels of malondialdehyde (MDA), hydrogen peroxide (H_2_O_2_), proline (Pro), total phenolic compounds (TPC), total flavonoids (TF), Na^+^ and Cl^-^ concentrations (in roots and leaves) and, to a lesser extent, K^+^ concentration (in roots and leaves), which indicates that higher levels of salinity induced significant levels of osmotic, ionic and oxidative stress. On the other hand, the biostimulant was most strongly related to root area (RA) [mainly to the area of very fine roots (VFRA), fine roots (FRA) and thin roots (TRA)], fresh weight of roots (FWr) and leaves (FWl), the water content in roots (WCr) and leaves (WCl), pigment contents, total chlorophyll (tChl) and carotenoids (Caro), calcium content in roots [Ca (r)] and leaves [Ca (l)], glycine betaine (GB) and, to a lesser extent, with mean root area (MRA), coarse root area (CRA), and total soluble sugars (TSS).

BALOX^®^ had a stimulating effect on the performance of the root system, which is the first organ exposed to the deleterious effects of salt and plays a critical role in maintaining water and nutrient uptake while preventing the influx of toxic ions under salt stress conditions. In addition, BALOX^®^ appeared to enhance the bioaccumulation of potassium; potassium metabolism is also crucial under salinity conditions but is often disrupted by excess salt in the substrate because its physicochemical properties are similar to those of sodium. Indeed, potassium is essential to maintain osmotic and nutritional balance under salinity stress and to support plant growth and tolerance. It seems that BALOX^®^ treatment also increased Ca^2+^ content, although to a lesser extent, as shown by the Ca barycentre located on the positive side of PC2, similar to that of BALOX^®^. Calcium is another vital compound under salt stress, as it plays a crucial role in mediating the salt stress response, apart from helping maintain cell membrane stability and cell wall structure.

## 4 Discussion

The negative impact of salt stress on various crops is well documented, and lettuce is particularly affected as it has a lower salinity threshold than many other vegetables, between 1.1 dS m^-1^ and 2.0 dS m^-1^ (Andriolo et al., 2005; Uenluekara et al., 2008). Values above 2.8 dS m^-1^ in irrigation water cause a significant reduction in the growth of commercial lettuce cultivars (Rosas et al., 2019), and at 5.1 dS m^-1^, its yield is reduced by half (Havlin et al., 2005). Our findings confirmed that the growth of lettuce plants was gradually reduced by increasing salinity, and root and leaf fresh weight decreased significantly in parallel with increasing salinity in the irrigation solution, as reported in previous studies on this species (Yildirim et al., 2011; Ekinci et al., 2012; Cristofano et al., 2023). This weight reduction was partly due to plant dehydration, as shown by the salt-induced decrease in root and leaf water content.

In our experiments, total chlorophyll and carotenoid concentrations also decreased with increasing external salinity, in agreement with the known fact that salt stress negatively affects the photosynthetic activity, limiting chlorophyll biosynthesis (Rizwan et al., 2015) and reducing the concentration of other components such as carotenoids and xanthophylls, as reported in many horticultural crops, including lettuce (Saha et al., 2010; Pérez-López et al., 2013; Al Hassan et al., 2015). The soluble salt poured into the substrate is in direct contact with the roots, causing a reduction in the osmotic potential and hindering the absorption of water and nutrients (Parihar et al., 2015). This results in changes in plant morphology and adverse effects on plant metabolism and biomass accumulation, leading to plant growth arrest at high NaCl concentrations (Shalhevet, 1993; Le Rudulier, 2005; Souri and Tohidloo, 2019). In addition, using the microrhizotron tubes and chamber that provide a non-destructive method to assess root development in real-time (Andersen et al., 2014), it was observed that salinity treatments also significantly inhibited root growth and reduced root area; at concentrations of 100 and 150 mM NaCl, more damage was observed in the form of burns, compromising various areas of the root, such as the root cap, apical meristem, elongation and root hair zones. Previous studies indicated that 60 mM NaCl strongly affected spinach’s and lettuce’s root elongation and vegetative growth (Kaya et al., 2002). Similar concentrations, 50 to 100 mM NaCl, also affected *Medicago sativa* and *Vicia faba*, mostly the thinnest roots and root hairs, which are essential in nutrient uptake. Inhibition of root growth is a typical response to limiting factors such as lack of water or nutrients, toxic compounds, compaction of the plant medium (substrate or soil), or salinity (Neumann and Römheld, 2002). These constraints are even more pronounced in modern horticultural cultivars that generally have more reduced and shallower root systems than their wild ancestors (Kerbiriou et al., 2013).

According to Byrt et al. (2018), a frequent consequence of salt stress is a reduction in the cell elongation rate, whereby root growth zones contract in the presence of salts, limiting water and nutrient uptake. Salt stress can also alter the nutritional balance because it generates antagonistic effects on the nutrients’ uptake and transport within the plant (Parihar et al., 2015). Salinity induces Na^+^ toxicity, as it competes with various cations, such as Ca^2+^, for binding sites in the root cell wall (Hoffmann et al., 2020), inhibiting the activity of specific ion transporters located in the plasma membrane (Lu et al., 2016). Moreover, high intracellular Na^+^ concentrations alter the expression of several genes and the activity of essential enzymes, leading to metabolic deficiencies (Petretto et al., 2019).

In the present study, Na^+^ and Cl^-^ concentrations increased significantly with increasing salinity in the roots and leaves of lettuce plants, whereas Ca^2+^ concentration decreased. However, K^+^ contents increased in parallel with rising salinity levels and were about 5-fold higher in leaves than in roots, indicating an active transport of K^+^ to the aerial part. Carillo et al. (2020) found similar responses in a study on two varieties of lettuce subjected to different concentrations of salts, reporting an increase in foliar K^+^ in one of the varieties. The active transport of K^+^ from roots to leaves appears as a relevant defence mechanism in lettuce, activated in response to salt stress conditions (Wang et al., 2013). It has been reported that relatively low levels of K^+^ compartmentalised in the cytosol can counteract the osmotic potential of the vacuole, minimising stress damage and restoring plant growth (Cuin et al., 2009; Annunziata et al., 2017).

To counteract osmotic imbalances, plants activate a series of mechanisms of osmotic adjustment, accumulating various types of compatible solutes, or osmolytes, amongst which proline, glycine betaine, and soluble sugars stand out (Al-Yasi et al., 2020). Proline has been reported in various crops as a marker of the level of stress experienced by plants (Arteaga et al., 2020; Alvarez et al., 2022). In our experiments, proline concentration increased in parallel to the concentration of NaCl, as previously reported for lettuce plants (Bulgari et al., 2019). Furthermore, the correlation matrix analysis and the PCA evaluation indicated a high correlation of this osmolyte with the levels of MDA and H_2_O_2_, all of them closely linked to the presence of Na^+^ and Cl^-^ ions, indicating that higher salinity levels caused higher oxidative stress in lettuce plants.

It has been reported that soluble sugars and glycine betaine concentrations increase under saline conditions in different plant species, including lettuce (Hayashi et al., 1997; Saneoka et al., 1999; Leyva et al., 2011). However, we detected a reduction in the concentrations of these osmolytes with increasing salinity. Total sugars and glycine betaine play multiple roles in plants, and these changes must be interpreted cautiously (Paradisone et al., 2015; Calone et al., 2022). A decrease in soluble sugars under saline conditions may be related to low carbohydrate availability, resulting from decreased photosynthesis (Goicoechea et al., 2005), which would also fit our results showing a reduction of photosynthetic pigment levels with increasing salinity.

Salt stress also leads to excessive formation of reactive oxygen species (ROS), which can cause oxidative damage to different biomolecules and complex cellular structures (DNA, RNA, membrane lipids, proteins), compromising their stability and thus the vital functions they perform in plants (Guo et al., 2019). Oxidative degradation of lipids and increases in free radicals were reported in lettuce plants subjected to salt stress (Leyva et al., 2011; Mahmoudi et al., 2011). In line with these findings, our results revealed that salinity significantly increased the concentration of MDA and H_2_O_2_, two markers of oxidative stress (Kumar et al., 2017), as previously found in lettuce plants subjected to a 100 mM NaCl treatment (Yildirim et al., 2015).

Plants employ various mechanisms to defend themselves against oxidative stress, involving the activation of several enzymatic and non-enzymatic antioxidant systems, including the synthesis and accumulation, for example, of phenolic compounds, flavonoids or carotenoids (Sachdev et al., 2021). Our results showed that the NaCl treatments increased the concentration of total phenolic compounds and flavonoids in romaine lettuce plants, as reported before by other authors (Mahmoudi et al., 2010; Carillo et al., 2020). It is well known that plants synthesise phenolic compounds, including flavonoids, in the absence of stress, as these compounds play multiple biological roles (Flores et al., 2022; Santander et al., 2022), but under salt stress conditions, these antioxidants may accumulate to higher levels, to alleviate the salt-driven increase of ROS in plant cells (Neocleous et al., 2014).

The treatment with the biostimulant BALOX^®^ did not modify the general responses of lettuce plants to salt stress discussed above. Thus, in parallel to increased NaCl concentration in the irrigation water, the growth of BALOX^®^-treated plants was inhibited, and root area, photosynthetic pigment levels, Ca^2+^ contents in roots and leaves, and soluble sugar and glycine betaine concentrations decreased. On the other hand, root and leaf Na^+^, Cl^-^ and K^+^ concentrations, as well as proline, MDA, H_2_O_2_, TPC and TF leaf contents, significantly increased with increasing salinity. However, the biostimulant improved all growth and biochemical parameters of the plants compared to those of the untreated controls. Other authors have also reported that biostimulants can significantly increase the fresh weight of plants and improve photosynthetic activity under salt-stress conditions in horticultural crops such as lettuce (Bulgari et al., 2019). Indeed, the concentration of photosynthetic pigments is a primary indicator of plant growth, directly related to the rate of photosynthesis (Ghorbani et al., 2018). BALOX^®^ helped boost significantly total chlorophyll and carotenoid concentrations under all tested conditions, including high salinity, compared to untreated plants. Other authors have reported an increase in carotenoid contents in baby lettuce treated with a protein hydrolysate-based biostimulant (Di Mola et al., 2020; Sabatino et al., 2021), which increased photosynthetic efficiency and plant productivity (Yakhin et al., 2017). This effect is attributed to the ability of such biostimulants to activate primary and secondary plant metabolism, as well as the expression of genes related to defence mechanisms and stress tolerance (Shahrajabian et al., 2021). Carotenoids are essential in the protection of the photosynthetic machinery and cell membranes, for the absorption and transfer of light energy to chlorophylls, and are also involved in the elimination of reactive oxygen species (ROS) by dissipating excess solar radiation (Das and Roychoudhury, 2014; Rodriguez-Concepcion et al., 2018).

BALOX^®^ application under all tested conditions, with and without salt, in addition to increasing root weight, helped expand the area of the root system, mainly that of the finest roots (below 2 mm diameter). It also had a protective and stimulating effect on the root hair zone compared to untreated plants. This effect on the root hair zone is highly relevant for nutrient absorption, and the development of plants’ aerial parts depends on the roots’ development and quality. These results are consistent with previous studies (Zuzunaga-Rosas et al., 2022; Zuzunaga-Rosas et al., 2023a) conducted with this biostimulant on tomatoes, where BALOX^®^ application increased root and leaf fresh weight even in the presence of high salt concentrations. The observed effect was related to the enhanced production of antioxidants such as carotenoids and auxin-like compounds, which induced root system growth. Root growth and root hair development are vital for plant development and adaptation to various environmental conditions (Han et al., 2023), leading to increased nutrient acquisition and stress resistance (Kawa, 2020; Li et al., 2022). Most plant species, including lettuce, can produce root hairs, which could be considered a small-scale sub-system of nutrient-seeking roots (Jungk, 2001). Root hairs are polarised tubular extensions arising behind the root elongation zone, formed by epidermal cells known as trichoblasts (Ridge, 1996). A crucial function of root hairs is to increase the root surface area and thus facilitate the absorption of nutrients by plants, in parallel to increasing root anchorage, interactions with the environment, and resistance to various types of stress (Peterson and Farquhar, 1996; Grierson et al., 2014; Vissenberg et al., 2020). In addition, root hairs contribute substantially to the acquisition of nutrients of low mobility in the soil and high demand by plants (Jungk, 2001), such as phosphorus and calcium. Several authors (Battacharyya et al., 2015; Colla et al., 2015) have suggested that the application of biostimulants based on plant protein hydrolysates externalise activities analogous to auxin, cytokinin and gibberellin-type phytohormones, and also intervene in the synthesis of photosynthetic pigments, secondary metabolites and antioxidant enzymes. A previous study on lettuce (Lucini et al., 2015) reported a significant increase in the absorption area as a result of changes in the morphology of the root system stimulated by the action of biostimulants, which could correspond to the activation of a mechanism of tolerance to salt stress (Tuteja, 2007).

The application of BALOX^®^ also induced a significant reduction of toxic ions, Na^+^ and Cl^-^, in plant tissues and favoured the accumulation of Ca^2+^ and K^+^, as it has also been reported in the presence of a biostimulant based on protein hydrolysates in the same species (Rouphael et al., 2022). The biostimulant effect could be mediated by activating the primary Na^+^ extrusion mechanism in plants, the plasma membrane H^+^-ATPase (Sussman, 1994). In addition, it has been reported that protein hydrolysates are also involved in nitrogen assimilation by stimulating the action of nitrate reductase and glutamine synthetase (Ertani et al., 2009). Furthermore, several authors have shown that glycine betaine, which is included in BALOX^®^ composition, reduced Na^+^ and Cl^-^ accumulation and increased Ca^2+^ and K^+^ concentrations in leaves (Habib et al., 2012; Khalifa et al., 2016).

In BALOX^®^-treated plants, leaf proline concentration did not vary with respect to the untreated controls in the absence of salt. However, it decreased significantly under all salinity conditions tested. We have previously assessed the biostimulant effects on tomato plants with similar results: a significant relative reduction of salt-induced proline accumulation compared to untreated plants (Zuzunaga-Rosas et al., 2023b). The same effect was observed for a different amino acids-based biostimulant, also in tomato (Gil-Ortiz et al., 2023). Such results agree with those of other authors (Cristofano et al., 2023), who also applied plant-derived biostimulants to lettuce. As mentioned above, proline is considered an excellent stress biomarker for many crops (Arteaga et al., 2020; Alvarez et al., 2022). Therefore, it could be concluded that these biostimulants alleviated the stress suffered by plants under high salinity conditions. However, this conclusion cannot be generalised since other reports showed the opposite effect, an increase in proline contents in biostimulant-treated plants with respect to untreated controls at the same salt concentration; for example, when lettuce plants were treated with different plant protein hydrolysates (Zuluaga et al., 2023), or applying a seaweed extract to tomato plants (Gil-Ortiz et al., 2023). Therefore, further research is needed to establish the participation of this osmolyte in biostimulants’ mechanisms of action.

We also observed that BALOX^®^ raised the mean concentrations of TSS and GB in the presence and absence of salt, although the differences with the values measured in plants not treated with the biostimulant were, in most cases, not statistically significant. This trend can be explained by the stimulation of photosynthesis by BALOX^®^, in the case of TSS, and by the inclusion of GB as one of the active components in the biostimulant formulation. Previous reports indicated that exogenous applications of GB stimulated the increase of endogenous GB and other osmolytes under salinity conditions (Wang et al., 2010; Khalifa et al., 2016).

The present study showed that applying the biostimulant to salt-stressed lettuce plants significantly reduced MDA and H_2_O_2_ contents compared to untreated controls. Since these compounds are reliable oxidative stress biomarkers (Kumar et al., 2017), it can be concluded that BALOX^®^ helped reduce the oxidative stress generated as a secondary effect of the salt stress treatments, probably improving the plants’ anti-oxidant defence mechanisms. We have previously reported the same effect of the biostimulant in tomato plants (Zuzunaga-Rosas et al., 2023a). Additional reports have been published showing reductions in the concentration of MDA and H_2_O_2_ by the application of other biostimulants (Ouhaddou et al., 2023) or exogenous GB in lettuce species (Giri, 2011; Yildirim et al., 2015). Moreover, there are also data supporting that biostimulants based on plant protein hydrolysates can enhance the antioxidant defence systems of different plant species, including lettuce (Rouphael et al., 2017; Giordano et al., 2022; El-Nakhel et al., 2023). The principal component analysis (PCA) performed in this study suggested that BALOX^®^-treated plants subjected to salt stress treatments responded similarly to control, non-stressed or less stressed plants, requiring lower antioxidant activity to reduce the deleterious effects of salinity.

## 5 Conclusions

Salt stress significantly inhibited the growth of lettuce plants in a concentration-dependent manner, reducing photosynthetic pigment contents and increasing the concentrations of toxic ions (Na^+^, Cl^-^) in roots and leaves (partly counteracted by the active transport of K^+^ from roots to the aboveground plant organs), while reducing those of Ca^2+^. The salt treatments also induced the synthesis and accumulation of proline in leaves for osmotic adjustment and generated oxidative stress, as shown by the increase in MDA and H_2_O_2_, two reliable oxidative stress biomarkers.

The treatment with the biostimulant BALOX^®^ did not modify the general responses of lettuce plants to salt stress; however, it improved all their growth and biochemical parameters compared to those of the untreated controls. Thus, BALOX^®^ increased the plants’ root and leaf fresh weight and water content, and the levels of chlorophylls and carotenoids while reducing Na^+^ and Cl^-^ and increasing Ca^2+^ contents in roots and leaves, in the presence and absence of salt. Moreover, the biostimulant also reduced the salt-induced increase in proline, MDA and H_2_O_2_ concentrations. Therefore, BALOX^®^ showed an apparent effect in reducing the osmotic, ionic and oxidative stress levels caused by elevated salinity in lettuce. Similar results have been previously reported for different biostimulants in this and other plant species. The novelty of the present work is that, combined with the physiological and biochemical analyses, we conducted a real-time observation of the effects of salinity and the biostimulant on the plant roots. This holistic approach allowed us to propose that the biostimulant effects are primarily mediated by improving plant nutrition, even under severe salt stress conditions, by protecting and stimulating the root absorption zone.

## Data availability statement

The raw data supporting the conclusions of this article will be made available by the authors, without undue reservation.

## Author contributions

JZ-R: Conceptualisation, Data curation, Formal analysis, Investigation, Methodology, Software, Visualisation, Writing – original draft, Writing – review & editing. RC: Data curation, Software, Writing – review & editing. DM: Formal analysis, Writing – review & editing. RS: Formal analysis, Writing – review & editing. SI-A: Conceptualisation, Funding acquisition, Methodology, Project administration, Resources, Supervision, Validation, Writing – review & editing. MB: Conceptualisation, Data curation, Funding acquisition, Methodology, Project administration, Resources, Supervision, Validation, Writing – review & editing. AF: Software, Validation, Writing – review & editing. HM-R: Conceptualisation, Funding acquisition, Methodology, Project administration, Resources, Supervision, Validation, Visualisation, Writing – review & editing. OV: Conceptualisation, Funding acquisition, Methodology, Project administration, Resources, Supervision, Validation, Visualisation, Writing – review & editing.
